# Factor Structure and Internal Consistency on a Reduced Version of the Revised Test of Need for Cognitive Closure

**DOI:** 10.3389/fpsyg.2021.813115

**Published:** 2022-01-14

**Authors:** Luis Carlos Jaume, Christian Schetsche, Marcelo Agustín Roca, Paula Quattrocchi

**Affiliations:** Instituto de Investigaciones, Facultad de Psicología, Universidad de Buenos Aires, Buenos Aires, Argentina

**Keywords:** need for cognitive closure, RT-NFC, factor structure, reduction, internal consistency

## Abstract

The need for cognitive closure is a construct postulated by Kruglanski that explains the motivational aspects which influence decision-making and its impact on the social environment. Initially, it was assessed through a unidimensional scale, later criticized for its poor satisfactory reliability and validity. Regarding these criticisms, Pierro and Kruglanski developed a new 14-item scale to measure two dimensions, which were not previously evaluated: urgency tendency and permanence tendency. Although the Revised Test of Need for Cognitive Closure is more economical in terms of assessment time, it would be optimal to develop a reduced test that can assess faster while maintaining validity and reliability. The present research aims to reduce the Revised Test of Need for Cognitive Closure scale to the Argentinian context. To this end, we worked on a non-experimental design, assessing this scale within a sample of 690 Argentinian university students (Women = 81.16%, Men = 18.84%), and proceeded to perform reliability, as well as confirmatory factor analysis, convergent validity, and factorial invariance analysis. The results indicate a bi-factorial structure of a Need for Cognitive Closure instrument with eight items and two dimensions: urgency tendency (α = 0.76) and permanence tendency (α = 0.64), suggesting good reliability in both of them. In addition, well convergent validity was checked with other validated instruments, and finally, the factor loadings were shown to be invariant. In conclusion, it was demonstrated the reliability and validity of reducing the Revised Test of Need for Cognitive Closure in our social environment.

## Introduction

Social cognition is a discipline of social psychology dedicated to the study of the interaction between thought processes and social interaction (Kruglanski, 2013). One theory from the perspective of social cognition that explores these psychological phenomena is the need for cognitive closure (NFC; Kruglanski, 1989), which enables not only a better understanding of how we reason and form judgments and attitudes but also to understand better how we relate and interact with people, how we function in groups and interact with groups other than our own.

The NFC is a theory that explains the motivational aspects of decision making, as well as the relationship between this aspect and the cognitive properties of the individual in the process of knowledge acquisition (Kruglanski, 2013). The construct represents a motivational tendency whose magnitude is determined by the benefits and costs of closure relative to the benefits and costs of non-closure (Roets et al., 2015). Thus, it is possible to understand NFC as the desire of individuals to seek and maintain an answer to a problem (Kruglanski et al., 2006).

Epistemic motivations are implicated in people’s tendency to be closed-minded or open-minded (Kruglanski et al., 2018). Various NFC researches have enabled a better understanding of how epistemic motivation is relevant for human knowledge formation, influencing a broad spectrum of cognitive and social phenomena (Kossowska et al., 2018). Kruglanski and Webster (1996) described motivation toward closure as a continuous variable, in which there is a high need to achieve closure at one end and a high need to avoid closure at the other end. These motivational tendencies, in turn, are defined as two continuous variables (DeBacker and Crowson, 2009). The first variable is associated with motivation toward nonspecific closure, ranging from a strong desire to obtain it to a strong desire to avoid it. On the other hand, the second variable is motivation toward specific closure, which ranges from a powerful need to possess it to a powerful need to avoid it (Kruglanski et al., 2018).

To measure NFC dispositionally, Webster and Kruglanski (1994) developed a scale, which provides, using 42 items with a six-point Likert-type format, the possibility of analyzing the construct in the adult population. The authors made this scale with just one dimension through five main aspects. However, Neuberg et al. (1997), in a review of the NFC scale, pointed out on the one hand that the scale showed little inter-item homogeneity, while on the other hand, a factor analysis revealed that the instrument had a multifactorial structure.

Since the study previously mentioned, Pierro and Kruglanski (2005) developed the Revised NFC Test (RT-NFC). Using a factorial analysis, they got a first-order factor composed of the NFC and two second-order factors: the urgency tendency and the permanence tendency (Jaume et al., 2015). Regarding the urgency tendency, this stipulates an inclination to quickly get information, which allows giving rise to closure, whereas the permanence tendency refers to maintaining closure, freezing the acquired knowledge (Roets, 2017). In addition, the RT-NFC specifies 14 items, being ostensibly narrower than the original scale with its 42 items (Pierro and Kruglanski, 2005). Finally, a significant correlation with the original test scores was demonstrated in the North American population (*r* = 0.92; *p* < 0.01) and in the Italian population (*r* = 0.93; *p* < 0.01) (Pierro and Kruglanski, 2005).

Need for cognitive closure is an individual construct that determines how people obtain and transmit information and judgments (Kruglanski et al., 2006). In this sense, NFC represents individual differences in the desire for a quick, definitive, and stable answer to a question (Kruglanski and Fishman, 2009). Thus, several studies demonstrate the relationship between NFC and intrapersonal and interpersonal communications, such as cognitive control (Szumowska and Kossowska, 2016), social cognition (Szumowska and Kossowska, 2017), and social behaviors (Brizi and Biraglia, 2021), among others. This construct also affects the process of knowledge construction as it is often influenced by external agents, considered as epistemic authorities, that modify individual judgments. Because of these external agents, individuals compensate for the possible presence of logical inconsistencies within their everyday lives, offering individuals a state of certainty and control over the shared reality (Kruglanski et al., 2009).

The NFC comes from the theory of motivated social cognition, which argues that cognitive activities are not exempt from a motivational basis (Kruglanski, 1989). Thus, for Kunda (1990), motivation is present in all the different phases of the cognitive process. These phenomena imply that the NFC impacts the social environment, demonstrating that decisions are not made in an aseptic and rational place outside all motivation but powerfully influenced by it (Kruglanski et al., 2013).

Concerning the last paragraph, there are studies which showed that the construct could serve an adaptive purpose, as it enables favoritism and loyalty within the group (Kruglanski et al., 2002), enhanced feelings of solidarity toward the in-group (Orehek et al., 2010), and stable transmission of cultural norms (Livi et al., 2015). Despite this, it also could be a factor leading to culturally considered negative behaviors; such as intergroup hostility (Dugas et al., 2018), authoritarianism (De Keersmaecker et al., 2017), sexism (Pica et al., 2018), and prejudice in general (Van Hiel et al., 2004; Dhont et al., 2011; Burke et al., 2017; Baldner et al., 2019). That is why the NFC scale has been consistently correlated with constructs such as Right-Wing Authoritarianism (RWA; De Keersmaecker et al., 2017; Makwana et al., 2018); and Social Dominance Orientation (SDO; Van Hiel et al., 2004; Dhont et al., 2011).

On the other hand, although the RT-NFC is shorter than the original, contemporary times, characterized by acceleration and immediacy, demand new and faster evaluation forms than the old times. The scales elaborated with a great length require too much time to be answered, usually generating exhaustion in the respondents (Grillo Canelo, 2013). Because of this, the answers may be contaminated.

Therefore, the present research aims to explore the possibility of developing a reduction of the RT-NFC scale, obtaining the same results as with the initial set of items and thus preserving its psychometric properties.

## Methodology

### Research Design

We worked based on a non-experimental, cross-sectional design. In addition, to fulfill the proposed objectives, an *ex post* facto prospective single-group study was carried out (Montero and León, 2007) since its purpose was to analyze the relationships between social dominance orientation, right-wing authoritarianism, and the participants’ need for cognitive closure.

### Sample

The sample was non-probabilistic, purposive, composed of 690 Argentinian university students (Women = 81.16%, Men = 18.84%). The reference population comprised students of 19 until 51 years of age, (Mage = 28.11; SDage = 5.73). Participants were students recruited during classes at the University of Buenos Aires. Participants did not receive any compensation for their participation. Responses were collected using paper and pencil. In addition, the inclusive criteria were composed of university students of the University of Buenos Aires who were more than 18 years old.

### Instruments

Data were collected through a self-administered completed by pencil and paper evaluation instrument, ensuring the anonymity of the participants. The instrument consisted of:

#### Revised Test for Need for Cognitive Closure

The version adapted to the Argentinian context was used to assess the construct (Jaume et al., 2015), which includes the suggestions made by Horcajo et al. (2011). The scale is an adaptation of the Revised NFC Test (RT-NFC; Pierro and Kruglanski, 2005) and is composed of 14 items grouped in the dimensions: Urgency Tendency (NFC-U; α = 0.79) (e.g., “En caso de incertidumbre, prefiero tomar una decisión inmediata, sea la que sea”; “Cuando me encuentro frente a varias alternativas potencialmente válidas, me decido a favor de una rápidamente y sin vacilaciones”; “Prefiero decidir de acuerdo con la primera solución disponible, en vez de considerar en detalle qué decisión debería tomar”) and Permanence Tendency (NFC-P; α = 0.70) (e.g., “Me siento incómodo cuando no logro dar una respuesta rápida a un problema al que me enfrento”; “Cualquier solución a un problema es mejor que permanecer en un estado de incertidumbre”; “Prefiero actividades en las que está siempre claro qué es lo que hay que hacer y cómo hay que hacerlo”), which together make up the NFC construct. The response format is Likert-type with six anchors, depending on the degree of agreement of the participants, with 1 = *Completamente en desacuerdo* and 6 = *Completamente de acuerdo*.

#### Social Dominance Orientation

The version adapted to the Argentinian context was used (Etchezahar et al., 2014). This scale is composed of 14 items grouped into the dimensions: Group Dominance (SDO-DG; α = 0.74) (e.g., “Para salir adelante en la vida, algunas veces es necesario pasar por encima de otros grupos de personas”; “Los grupos superiores deberían dominar a los grupos inferiores”) and Opposition to equality (SDO-OI; α = 0.83) (e.g., “Habría menos problemas si tratáramos a los diferentes grupos de manera más igualitaria”; “La igualdad entre grupos de personas debería ser nuestro ideal”), which together make up the SDO construct (α = 0.85). The response format is Likert-type with five anchors, depending on the degree of agreement of the participants, with 1 = *Completamente en desacuerdo* and 5 = *Completamente de acuerdo*.

#### Right Wing Authoritarianism

A reduced version of the right wing authoritarianism (RWA) scale composed of a six-item dimension was used to assess the construct (e.g., “Nuestro país necesita un líder poderoso que pueda enfrentar a los extremistas e inmorales que actualmente prevalecen en nuestra sociedad”; “Nuestros ancestros debieron ser más honrados por la forma en que construyeron esta sociedad, por ello, es necesario poner fin a las fuerzas que la están destruyendo”; “Los hechos muestran que debemos ser más duros con el crimen y la inmoralidad sexual con el fin de mantener la ley y el orden”), adapted and validated to the local context (Etchezahar et al., 2011) with a Cronbach’s alpha of (α = 0.74). The response format of the scale is Likert-type with five anchors according to the degree of agreement or disagreement with each statement, with 1 = *Totalmente en desacuerdo* and 5 = *Totalmente de acuerdo.*

#### Sociodemographic Variables

An *ad hoc* questionnaire was developed to collect the following variables: sex, age, and place of residence of the participants.

### Procedure

The university students who took part in this research participated voluntarily and anonymously after giving their consent. They were also informed that the data disclosed would be used exclusively for academic-scientific purposes. All this was under National Law 25.326 on the protection of personal data.

### Data Analysis

The robust Minimum Covariance Determinant test of Leys et al. (2018) was performed to detect multivariate outliers, using the MASS package of Venables and Ripley (2002). Also, multivariate normality calculation was performed with the MVN package of Korkmaz et al. (2014). Confirmatory factor analysis and factorial invariance analysis were performed with the help of the Lavaan package of Rosseel (2012). On the other hand, correlations calculation and descriptive statistics were performed with the psych package by Revelle (2019). The said packages are part of the R Core Team (2020). Finally, the probability value *p* ≤ 0.05 was used for all calculations performed.

## Results

### Outliers and Multivariate Normality

We performed the robust Minimum Covariance Determinant test of Leys et al. (2018) to detect multivariate outliers. As a result, 13 values were classified as severe outliers, so they were eliminated from the sample, leaving it at 677.

Following Fokkema and Greiff (2017) suggestions, we divided the sample in two: With sample A (*n* = 338), we carried out the exploratory factor analysis and item reduction, and with sample B (*n* = 339), we carried out the convergent validity and factorial invariance analysis.

The non-existence of multicollinearity was ascertained and, through the use of the Mardia (1970) test, it was found that the data did not represent multivariate normality, so we proceeded to evaluate the model fits through the Weighted Least Squares with Means and Variances adjusted (WLSMV), thus complying with the review of the recent literature discussion on the CFA estimator (Li, 2016).

According to the indications of Hu and Bentler (1999), we can consider a model as adequate when its fit takes the following values: Standardized Root Mean Square Residual (SRMR) ≤ 0.08, Root Mean Square Error of Approximation (RMSEA) ≤ 0.06, Comparative Fit Index (CFI) ≥ 0.95, Tucker Lewis Index (TLI) ≥ 0.95. Although other authors, for example, Marsh et al. (2004), highlight the importance of evaluating the complexity of the model and the sample size. Following the authors’ indications, the ideal fit indices for evaluating a simple model consisting of the SRMR and the χ^2^/df for the case of the sample size of the present study.

### Purification Measures

Through an exploratory factorial analysis, the bi-factorial structure of the instrument was ascertained, which is in line with the study conducted by Jaume et al. (2015). Based on this, the first confirmatory factor analysis was performed with its 14 original items.

Based on suggestions for scale development (DeVellis, 2017), items were eliminated that (a) by inspection of modification indices did not load more on their desired factor; (b) exhibited a large number of standardized residuals > 2.58 with other items; (c) had standardized factor loadings < 0.50; (d) were highly redundant in terms of wording with other items; and (e) had extremely high standardized factor loadings (>0.90). To preserve as much content coverage as possible (Smith et al., 2000), we analyzed the deleted items and noted that no content domains were removed from the main measure. Thus, the RT-NFC was reduced to 8 items and, using the method proposed by Saris et al. (2009), residual covariances were created for those items loading on the same factor so that the psychometric characteristics could be substantially improved.

After the purification measure, the item assignment is as follows. Urgency: (Item 1) Cuando estoy frente a varias alternativas potencialmente válidas, me decido a favor de una rápidamente y sin dudarlo, (Item 3) En situaciones de incertidumbre, prefiero tomar decisiones rápidas. (Item 5) Suelo tomar decisiones rápidas y sin pensar demasiado. (Item 7) Me gusta tomar decisiones rápidas. Permanence: (Item 2) Me siento muy incómodo cuando las cosas a mi alrededor no están en su sitio. (Item 4) Prefiero estar con personas que tienen las mismas ideas y gustos que yo. (Item 6) Me gustan más las actividades en las que está siempre claro qué es lo que hay que hacer y cómo hay que hacerlo. (Item 8) Prefiero cosas a las que estoy acostumbrado que aquéllas que no conozco y no puedo predecir.

Since high correlations were found between both dimensions, a second-order model and a uni-factor structure were tested. Table 1 shows all the models developed. On the one hand, the original instrument, the RT-NFC-14-2F with two dimensions and 14 items, the NFC-8-2F with two dimensions and eight items, the NFC-8-SO with a second-order structure and eight items, and the NFC-8-1F with a single dimension and eight items. Although the uni-factor model obtained a superior model fit, it should be noted that the fits of the eight-item, two-factor model were also found to be in a suitable range according to the classification of Hu and Bentler (1999) and Marsh et al. (2004). Next, we assessed in sample A the correlations between the NFC-U factor of RT-NFC-14-2F and RT-NFC-8-2F (*r* = 0.952, *p* < 0.001) and, subsequently, between the NFC-U factor of RT-NFC-14-2F and RT-NFC-8-2F (*r* = 0.900, *p* < 0.001).

**TABLE 1:** Model adjustments of psychometric instruments, sample B (*N* = 339).

	χ^2^ WLSMV	df	χ^2^/df	*p*	RMSEA (90% CI)	SRMR	CFI	TLI
RT-NFC-14-2F	155.574	76	2.047	0.000	0.056 (0.043–0.068)	0.061	0.897	0.877
RT-NFC-8-2F	20.731	17	1.219	0.239	0.025 (0.000–0.058)	0.034	0.989	0.982
RT-NFC-8-SO	20.731	17	1.219	0.239	0.025 (0.000–0.058)	0.034	0.989	0.982
RT-NFC-8-1F	7.176	10	0.718	0.709	0.000 (0.000–0.045)	0.017	1.000	1.023

*F, factor; SO, second order; χ^2^ WLSMV – Scaled Chi-Square using Weighted Least Squares with Means and Variances adjusted; df, degrees of freedom; RMSEA, root mean square error of approximation; SRMR, standardized root mean square residual; CFI, comparative fit index; and TLI, tucker lewis index.*

### Convergent Validity

The convergent validity of the two-factor version was tested to assess whether the use of both dimensions would be justified, taking into account the favorable psychometric values of the uni-factor and bi-factor versions of the short form. For this analysis, Pearson correlations of NFC-U and NFC-P with SDO-DG, SDO-OI, and RWA were considered. Table 2 shows the NFC-P factor has a correlation of medium value with RWA, while the correlation between NFC-U and RWA is a low value (Cohen, 1988). Comparing the correlations between the short and long versions of the instrument had with RWA and SDO, it is observed that these correlations were found to be in similar ranges.

**TABLE 2:** Pearson correlations between RWA and SDO and the dimensions of the RT-NFC-14-2F (sample A), and RT-NFC-8-2F (sample B).

	RT-NFC-14-2F, sample A				
RT-NFC-8-2F, sample B	RWA	SDO DG	SDO OI	NFC-U	NFC-P
RWA	1	0.43**	–0.07	0.18**	0.36**
SDO-DG	0.48**	1	−0.36**	0.21**	0.23**
SDO-OI	–0.03	−0.34**	1	–0.08	–0.04
NFC-U	0.16**	0.29**	–0.05	1	0.29**
NFC-P	0.32**	0.33**	–0.03	0.22**	1

Sample A n = 338; sample B n = 339; ** Correlation is significant at the 0.01 level (bilateral).

### Factorial Invariance

A multiple-group analysis was conducted about gender to analyze whether the model represented factorial invariance. Table 3 shows the results of the analysis multiple-group, in which CFI and RMSEA changes were used to assess factorial invariance. It was found to be within appropriate ranges with ΔCFI ≥ −0.01 according to Cheung and Rensvold (2002) and ΔRMSEA ≥ 0.015 according to Chen (2007).

**TABLE 3:** Model fitting and model comparison for genders (*n* = 339).

Model	Model adjustment				Model comparison				
	χ^2^ WLSMV	df	RMSEA	CFI	Δχ^2^ WLSMV	Δdf	*p*	ΔRMSEA	ΔCFI
M1: Config.	36.220	34	0.020	0.992					
M2: Weak	38.544	40	0.017	1.000	2.324	6	0.888	−0.003	0.008
M3: Strong	45.308	46	0.017	1.000	6.764	6	0.343	0.000	0.000
M4: Strict	58.933	54	0.030	0.991	13.625	8	0.092	0.013	−0.009

*χ^2^ WLSMV – Scaled Chi-Square using Weighted Least Squares with Means and Variances adjusted; df, degrees of freedom; RMSEA, root mean square error of approximation; and CFI, comparative fit index.*

### Descriptive Statistics of the Psychometric Instruments

Internal consistencies of RT-NFC-14-2F (sample A) were α = 0.84 for NFC-U and α = 0.73 for NFC-P. According to the Spearman–Brown formula (Nunally and Bernstein, 1994), the estimated reliability of the short form was α = 0.66 for NFC-U and α = 0.50 for NFC-P. As shown in Table 4, the values obtained by RT-NFC-8-2F were above these. Although the internal consistency of the NFC-P was found to be slightly below the value suggested by Smith et al. (2000) (α > 0.70), it can be noted that other authors, e.g., Hinton (2014), consider an internal consistency of α > 0.50 as adequate.

**TABLE 4:** Descriptive statistics and internal consistencies of RT-NFC, RWA, and SDO (*N* = 339).

	M	SD	Mdn	Min	Max	Asymmetry	Kurtosis	Q 0.25	Q 0.50	Q 0.75	α
RWA	15.29	5.82	16.00	6.00	30.00	0.02	–0.85	10.00	16.00	20.00	0.83
SDO-DG	9.69	3.35	9.00	5.00	21.00	0.46	–0.30	7.00	9.00	12.00	0.62
SDO-OI	20.80	3.064	21.00	7.00	25.00	–0.80	0.16	19.00	21.00	24.00	0.77
NFC-U	2.18	0.78	2.00	1.00	5.00	0.58	0.27	1.75	2.00	2.75	0.76
NFC-P	2.92	0.81	3.00	1.00	4.75	–0.13	–0.33	2.25	3.00	3.50	0.64

*M, mean; SD, standard deviation; Mdn, median; Min, minimum; Max, maximum; Q, quartile; and α, Cronbach’s alphas.*

## Discussion

In the present study, a reduction of the RT-NFC scale was attempted. The results obtained show that an NFC model with eight items is within a suitable range according to the classification of Hu and Bentler (1999) and Marsh et al. (2004). In addition, it is a reliable instrument for the Argentinian context, since the NFC-U obtained an α = 0.76 (see Table 4), which demonstrates good reliability similar to both the research of Horcajo et al. (2011) and Jaume et al. (2015), while on the other hand, the NFC-P presents an α = 0.64 (see Table 4), which is an acceptable value and shows slightly below the study of Horcajo et al. (2011; α = 0.70). Likewise, we were able to demonstrate a favorable analysis in terms of its bifactoriality (see Table 1), thus corroborating the data obtained by both Pierro and Kruglanski (2005) and Horcajo et al. (2011).

The correlations of the NFC with the other instruments used were significant, which also demonstrated the convergence validity of this model. Also, the association between NFC, SDO, and RWA has been shown (see Table 2). Initially, a positive relationship was found between NFC-U and NFC-P, and RWA, as well as between NFC-U and NFC-P, and SDO-DG, and a correlation between these instruments could be affirmed as in numerous investigations (e.g., Van Hiel et al., 2004; Dhont et al., 2011; De Keersmaecker et al., 2017; Makwana et al., 2018). Thus, it is possible to conclude that there is a relationship between attitudinal aspects with motivational aspects of individuals.

Additionally, for greater robustness to this study, the factorial invariance was evaluated, which shows to be within the appropriate ranges with a ΔCFI ≥ −0.01 according to Cheung and Rensvold (2002) and a ΔRMSEA ≥ 0.015 according to Chen (2007), suggesting that the loading factors are invariant between the groups of men and women.

Even though the practice of shortening measurement scales still often lacks methodological rigor, we followed the theoretical model, and we tested the fit model. Furthermore, a shortened version, the RT NFC-8, possesses similar properties to the original and offers the advantage of brevity. To do this, we complied with the methodology to develop new CMSs (composite measurement scales; Goetz et al., 2013), including consideration of different psychometric properties.

To improve the shortening process and the validity of our resulting RT NFC-8 scale, we follow the six steps for new CMSs (Goetz et al., 2013). Firstly, we documented the validity of the original RT-NFC and the aim of its shortening. Secondly, we took the conceptual model into account. In the third and fourth steps, we preserved the content validity and the psychometric properties. Fifthly, we documented the reasons for item selection. Last but not least, we validated the short-form in an independent sample. In this sense, our results demonstrate the possibility of using an 8-item NFC, which could increase the studies in Social Psychology and the possible search for relationships with other scales.

### Limitations

Our results indicate the possibility of evaluating shortly and quickly the NFC construct in the Argentinian context. However, the study has limitations that can be overcome in future research, such as using a sample of university students that barely represents the population. In turn, this is related to the fact that the sample drawn is from a white, educated, industrialized, wealthy, and democratic (WEIRD) population. This reliance on a limited population of participants poses a threat to the external validity of our results. In this sense, it would be interesting to add samples from other social settings to increase the representativeness of the results.

## Conclusion

Our goal with this study was to reduce the RT-NFC scale which, would allow for greater ease of use, as well as a shortening the time of taking it. The burden of long scales and the increasing need for multiple instruments in the same study have created a need to shorten measurement scales. In this paper, we tried to reduce the number of items of the scale RT NFC while we pretended to preserve or improve its psychometric properties, generating a shorter and faster test to evaluate the NFC construct.

This tool will allow us to understand individuals’ motivational aspects and also the social environment where they belong. That’s how some individuals become themselves as epistemic authorities for others, generating and or shaping people’s opinions. Adherence to the perspective of authority epistemic compensates for the possible presence of logical inconsistencies within our everyday life, offering individuals a state of certainty and control over the shared reality. Although the decrease of uncertainty can be positive in some situations, in other cases, it generates harmful effects in the social environment. It could be seen, for example, in the case of following authoritarian leaders who contribute to rise intergroup conflicts and undermine democratic attitudes. Another phenomenon where the pros and cons of the NFC can be seen is prejudice, where it is convenient to have a crystallized knowledge that serves to act in a moment of emergency without hesitation. Nevertheless, in everyday conditions where survival is not at risk, a high NFC can collaborate and influence the increase of prejudice in its various manifestations (e.g., sexism, xenophobia, racism, anti-Semitism). For this reason, the evaluation of the NFC can be used to monitor and, if necessary, reduce these behaviors.

Finally, although the results point to the use of this reduced scale in the Argentinian context, there is still a long way to go to understand whether this tool can be implemented in other contexts different from the one in which we have worked.

## Data Availability Statement

The raw data supporting the conclusions of this article will be made available by the authors, without undue reservation.

## Ethics Statement

The studies involving human participants were reviewed and approved by the Instituto de Investigaciones, Facultad de Psicología. The patients/participants provided their written informed consent to participate in this study.

## Author Contributions

LJ: substantial contributions to the conception or design of the work and the acquisition, analysis or interpretation of data for the work. CS: acquisition, analysis or interpretation of data for the work. MR and PQ: drafting the work or revising it critically for important intellectual content. Agree to be accountable for all aspects of the work in ensuring that questions related to the accuracy or integrity of any part of the work are appropriately investigated and resolved. All authors contributed to the article and approved the submitted version.

## Conflict of Interest

The authors declare that the research was conducted in the absence of any commercial or financial relationships that could be construed as a potential conflict of interest.

## Publisher’s Note

All claims expressed in this article are solely those of the authors and do not necessarily represent those of their affiliated organizations, or those of the publisher, the editors and the reviewers. Any product that may be evaluated in this article, or claim that may be made by its manufacturer, is not guaranteed or endorsed by the publisher.

## References

[B1] Baldner C. Jaume L. C. Pierro A. Kruglanski A. W. (2019). The epistemic bases of prejudice: the role of need for cognitive closure. *TPM Test. Psychom. Methodol. Appl. Psychol.* 26 447–461. 10.4473/TPM26.3.9

[B2] Brizi A. Biraglia A. (2021). Do I have enough food?” How need for cognitive closure and gender impact stockpiling and food waste during the COVID-19 pandemic: a cross-national study in India and the United States of America. *Pers. Individ. Differ.* 168:110396. 10.1016/j.paid.2020.110396 32982000 PMC7501171

[B3] Burke S. E. Dovidio J. F. LaFrance M. Przedworski J. M. Perry S. P. Phelan S. M. (2017). Beyond generalized sexual prejudice: need for closure predicts negative attitudes toward bisexual people relative to gay/lesbian people. *J. Exp. Soc. Psychol.* 71 145–150. 10.1016/j/jesp.2017.02.003 28983126 PMC5624340

[B4] Chen F. F. (2007). Sensitivity of goodness of fit indexes to lack of measurement invariance. *Struct. Equ. Model.* 14 464–504. 10.1080/10705510701301834

[B5] Cheung G. W. Rensvold R. B. (2002). Evaluating goodness-of-fit indexes for testing measurement invariance. *Struct. Equ. Model.* 9 233–255. 10.1207/S15328007SEM0902_5 33486653

[B6] Cohen J. (1988). *Statistical Power Analysis for the Behavioral Sciences*, 2nd Edn. Milton Park: Routledge.

[B7] De Keersmaecker J. Roets A. Dhont K. Van Assche J. Onraet E. Van Hiel A. (2017). Need for closure and perceived threat as bases of right-wing authoritarianism: a longitudinal moderation approach. *Soc. Cogn.* 35 433–449. 10.1521/soco.2017.35.4.433

[B8] DeBacker T. K. Crowson H. M. (2009). The influence of need for closure on learning and teaching. *Educ. Psychol. Rev.* 21 303–323. 10.1007/s10648-009-9111-1

[B9] DeVellis R. F. (2017). *Scale Development: Theory and Applications*, 4th Edn. Thousand Oaks, CA: Sage Publications.

[B10] Dhont K. Roets A. Van Hiel A. (2011). Opening closed minds: the combined effects of intergroup contact and need for closure on prejudice. *Pers. Soc. Psychol. Bull.* 37 514–528. 10.1177/0146167211399101 21343438

[B11] Dugas M. Schori-Eyal N. Kruglanski A. W. Klar Y. Touchton-Leonard K. McNeill A. (2018). Group-centric attitudes mediate the relationship between need for closure and intergroup hostility. *Group Process. Intergroup Relat.* 21 1155–1171. 10.1177/1368430217699462

[B12] Etchezahar E. Cervone N. Biglieri J. Quattrocchi P. Prado-Gascó V. (2011). Adaptación y validación de la versión reducida de la escala de autoritarismo de derechas (RWA) al contexto argentino. *Anu. Invest.* 18 237–242.

[B13] Etchezahar E. Prado-Gascó V. Jaume L. Brussino S. (2014). Validación argentina de la Escala de Orientación a la Dominancia Social. *Rev. Latinoam. Psicol.* 46 35–43. 10.1016/S0120-0534(14)70004-4

[B14] Fokkema M. Greiff S. (2017). How performing PCA and CFA on the same data equals trouble. *Eur. Psychol. Assess.* 33 399–402. 10.1027/1015-5759/a000460

[B15] Goetz C. Coste J. Lemetayer F. Rat A. C. Montel S. Recchia S. (2013). Item reduction based on rigorous methodological guidelines is necessary to maintain validity when shortening composite measurement scales. *J. Clin. Epidemiol.* 66 710–718. 10.1016/j.jclinepi.2012.12.015 23566375

[B16] Grillo Canelo N. M. (2013). *Construcción y Validación de una Herramienta de Gestión Para Evaluar la Cultura de Seguridad en Entornos Industriales* Doctoral dissertation. Barcelona: Universitat Ramon Llull.

[B17] Hinton P. (2014). *SPSS Explained.* Milton Park: Routledge, 10.4324/9781315797298

[B18] Horcajo J. Díaz D. Gandarillas B. Briñol P. (2011). Adaptación al castellano del Test de Necesidad de Cierre cognitivo. *Psicothema* 23 864–870. 22047885

[B19] Hu L. Bentler P. M. (1999). Cutoff criteria for fit indexes in covariance structure analysis: conventional criteria versus new alternatives. *Struct. Equ. Model.* 6 1–55. 10.1080/10705519909540118

[B20] Jaume L. Cervone N. Biglieri J. Quattrocchi P. (2015). Propiedades psicométricas del Test revisado de Necesidad de cierre cognitivo (TR-NCC) en una muestra de estudiantes de la Universidad de Buenos Aires. *Invest. Psicol.* 20 55–60.

[B21] Korkmaz S. Goksuluk D. Zararsiz G. (2014). MVN: an r package for assessing multivariate normality. *R J.* 6 151–162. 10.32614/RJ-2014-031

[B22] Kossowska M. Szumowska E. Dragon P. Jaśko K. Kruglanski A. W. (2018). Disparate roads to certainty processing strategy choices under need for closure. *Eur. Rev. Soc. Psychol.* 29 161–211. 10.1080/10463283.2018.1493066

[B23] Kruglanski A. W. (1989). *Lay Epistemics and Human Knowledge.* Berlin: Springer.

[B24] Kruglanski A. W. (2013). *The Psychology of Closed Mindedness.* East Sussex: Psychology Press, 10.4324/9780203506967

[B25] Kruglanski A. W. Fishman S. (2009). Psychological factors in terrorism and counterterrorism: individual, group, and organizational levels of analysis. *Soc. Issues Policy Rev.* 3 1–44. 10.1111/j.1751-2409.2009.01009.x

[B26] Kruglanski A. W. Webster D. M. (1996). Motivated closing of the mind: “Seizing” and “freezing.”. *Psychol. Rev.* 103 263–283. 10.1037/0033-295X.103.2.263 8637961

[B27] Kruglanski A. W. Bélanger J. J. Gelfand M. Gunaratna R. Hettiarachchi M. Reinares F. (2013). Terrorism—A (self) love story: redirecting the significance quest can end violence. *Am. Psychol.* 68 559–575. 24128318 10.1037/a0032615

[B28] Kruglanski A. W. Dechesne M. Orehek E. Pierro A. (2009). Three decades of lay epistemics: the why, how, and who of knowledge formation. *Eur. Rev. Soc. Psychol.* 20 146–191. 10.1080/10463280902860037

[B29] Kruglanski A. W. Jasko K. Milyavsky M. Chernikova M. Webber D. Pierro A. (2018). Cognitive consistency theory in social psychology: a paradigm reconsidered. *Psychol. Inq.* 29 45–59. 10.1080/1047840X.2018.1480619

[B30] Kruglanski A. W. Pierro A. Mannetti L. De Grada E. (2006). Groups as epistemic providers: need for closure and the unfolding of group-centrism. *Psychol. Rev.* 113 84–100. 16478302 10.1037/0033-295X.113.1.84

[B31] Kruglanski A. W. Shah J. Y. Pierro A. Mannetti L. (2002). When similarity breeds content: need for closure and the allure of homogeneous and self-resembling groups. *J. Pers. Soc. Psychol.* 83 648–662. 10.1037/0022-3514.83.3.648 12219860

[B32] Kunda Z. (1990). The case for motivated reasoning. *Psychol. Bull.* 108 480–498. 10.1037/0033-2909.108.3.480 2270237

[B33] Leys C. Klein O. Dominicy Y. Ley C. (2018). Detecting multivariate outliers: use a robust variant of the Mahalanobis distance. *J. Exp. Soc. Psychol.* 74 150–156. 10.1016/j.jesp.2017.09.011

[B34] Li C.-H. (2016). Confirmatory factor analysis with ordinal data: comparing robust maximum likelihood and diagonally weighted least squares. *Behav. Res. Methods* 48 936–949. 10.3758/s13428-015-0619-7 26174714

[B35] Livi S. Kruglanski A. W. Pierro A. Mannetti L. Kenny D. A. (2015). Epistemic motivation and perpetuation of group culture: effects of need for cognitive closure on trans-generational norm transmission. *Organ. Behav. Hum. Decis. Process.* 129 105–112. 10.1016/j.obhdp.2014.09.010

[B36] Makwana A. P. Dhont K. Akhlaghi-Ghaffarokh P. Masure M. Roets A. (2018). The motivated cognitive basis of transphobia: the roles of right-wing ideologies and gender role beliefs. *Sex Roles* 79 206–217. 10.1007/s11199-017-0860-x

[B37] Mardia K. V. (1970). Measures of multivariate skewness and kurtosis with applications. *Biometrika* 57 519–530. 10.1093/biomet/57.3.519

[B38] Marsh H. W. Hau K.-T. Wen Z. (2004). In search of golden rules: comment on hypothesis-testing approaches to setting cutoff values for fit indexes and dangers in overgeneralizing Hu and Bentler’s (1999) Findings. *Struct. Equ. Model.* 11 320–341. 10.1207/s15328007sem1103_2

[B39] Montero I. León O. G. (2007). A guide for naming research studies in psychology. *Int. J. Clin. Health Psychol.* 7 847–862.

[B40] Neuberg S. L. Judice T. N. West S. G. (1997). What the Need for Closure Scale measures and what it does not: toward differentiating among related epistemic motives. *J. Pers. Soc. Psychol.* 72 1396–1412. 10.1037/0022-3514.72.6.1396

[B41] Nunally J. C. Bernstein I. H. (1994). *Psychometric Theory.* New York, NY: McGraw-Hill.

[B42] Orehek E. Fishman S. Dechesne M. Doosje B. Kruglanski A. W. Cole A. P. (2010). Need for closure and the social response to terrorism. *Basic Appl. Soc. Psychol.* 32 279–290. 10.1080/01973533.2010.519196

[B43] Pica G. Pierro A. Pellegrini V. De Cristofaro V. Giannini A. Kruglanski A. W. (2018). “Keeping in mind the gender stereotype”: the role of need for closure in the retrieval-induced forgetting of female managers’ qualities. *Cogn. Process.* 19 363–373. 10.1007/s10339-018-0864-7 29779169

[B44] Pierro A. Kruglanski A. W. (2005). *Revised Need for Cognitive Closure Scale. Unpublished Manuscript.* La Sapienza: Università di Roma.

[B45] R Core Team (2020). *R: A Language and Environment for Statistical Computing.* Vienna: R Core Team.

[B46] Revelle W. (2019). *psych: Procedures for Personality and Psychological Research.* Available online at: https://cran.r-project.org/package=psych Version = 1.9.12 (accessed November 04, 2020).

[B47] Roets A. (2017). “Three decades of need for closure research – about epistemic goals and (not) means,” in *The Motivation-Cognition Interface: From the lab to the Real World: A Festschrift in Honor of Arie W. Kruglanski*, eds Kopetz C. E. Fishbach A. (New York, NY: Routledge), 39–55.

[B48] Roets A. Kruglanski A. W. Kossowska M. Pierro A. Hong Y. (2015). The motivated gatekeeper of our minds. *Adv. Exp. Soc. Psychol.* 52 221–283. 10.1016/bs.aesp.2015.01.001

[B49] Rosseel Y. (2012). lavaan: an R package for structural equation modeling. *J. Stat. Softw.* 48 1–36. 10.18637/jss.v048.i02

[B50] Saris W. E. Satorra A. van der Veld W. M. (2009). Testing structural equation models or detection of misspecifications? *Struct. Equ. Model.* 16 561–582. 10.1080/10705510903203433

[B51] Smith G. T. McCarthy D. M. Anderson K. G. (2000). On the sins of short-form development. *Psychol. Assess.* 12 102–111. 10.1037/1040-3590.12.1.102 10752369

[B52] Szumowska E. Kossowska M. (2016). Need for closure and multitasking performance: the role of shifting ability. *Pers. Individ. Differ.* 96 12–17. 10.1016/j.paid.2016.02.055

[B53] Szumowska E. Kossowska M. (2017). Motivational rigidity enhances multitasking performance: the role of handling interruptions. *Pers. Individ. Differ.* 106 81–89. 10.1016/j.paid.2016.10.040

[B54] Van Hiel A. Pandelaere M. Duriez B. (2004). The impact of need for closure on conservative beliefs and racism: differential mediation by authoritarian submission and authoritarian dominance. *Pers. Soc. Psychol. Bull.* 30 824–837. 10.1177/0146167204264333 15307224

[B55] Venables W. N. Ripley B. D. (2002). *Modern Applied Statistics with S.* New York, NY: Springer. 10.1007/978-0-387-21706-2

[B56] Webster D. M. Kruglanski A. W. (1994). Individual differences in need for cognitive closure. *J. Pers. Soc. Psychol.* 67 1049–1062. 10.1037/0022-3514.67.6.1049 7815301

